# Nitrogen deprivation differentially alters lipid accumulation and pyrolysis-derived volatile profiles in three microalgal species

**DOI:** 10.1186/s40643-026-01114-4

**Published:** 2026-08-17

**Authors:** Carlos Vicente Garza-León, Hans Christian Correa-Aguado, Gloria Viviana Cerrillo-Rojas, Roberto Rico Martínez, Omar Gómez-García, Iván Salgado-Transito

**Affiliations:** 1 Centro de Investigaciones en Óptica, A.C., Prol. Constitución 607, Fracc. Reserva Loma Bonita, 20200 Aguascalientes, México; 2 Unidad Profesional Interdisciplinaria de Ingeniería Campus Zacatecas (UPIIZ), Instituto Politécnico Nacional, 98160 Zacatecas, México; 3 Unidad Académica de Ciencias Químicas, Universidad Autónoma de Zacatecas, 98000 Zacatecas, México; 4 Departamento de Química, Centro de Ciencias Básicas, Universidad Autónoma de Aguascalientes, Avenida Universidad 940, 20131 Aguascalientes, México; 5 Departamento de Química Orgánica, Escuela Nacional de Ciencias Biológicas, Instituto Politécnico Nacional, Prolongación de Carpio y Plan de Ayala S/N, Col. Santo Tomás, 11340 Ciudad de México, México; 6 Garzada-General Industry Services and Projects, Sierra Nevada, 20120 Aguascalientes, México

**Keywords:** Nitrogen stress, Microalgal biomass, Lipid metabolism, Thermochemical conversion, Protein–protein interaction networks (PPI/STRING), Bioenergy

## Abstract

Nitrogen deprivation (N−) is widely used to induce lipid accumulation in microalgae. However, its impact on the relationship between lipid accumulation and thermochemical behavior remains poorly understood. In this study, a comparative experimental approach was used to evaluate the effects of nitrogen availability on growth kinetics, lipid accumulation, and pyrolysis-derived volatile compounds in *Chlorella vulgaris*, *Scenedesmus obliquus*, and *Nannochloropsis oculata*. Under nitrogen˗replete conditions (N +), all species showed higher growth rates, whereas N− reduced proliferation but increased lipid content by 5.0-fold in *C. vulgaris*, 2.4-fold in *S. obliquus*, and 1.8-fold in *N. oculata*. Py-GC/MS analysis revealed that N− shifted the pyrolysis profile toward lipid˗derived compounds, particularly C16˗C18 fatty acids, long˗chain alcohols, and hydrocarbons. In *C. vulgaris*, oleic acid (22.3%) and alcohol derivatives dominated; in contrast, N + conditions exhibited a more heterogeneous profile, including aromatics and sterols. *N. oculata* maintained a predominance of lipid-derived compounds, with N− associated with a higher contribution of oxygenated compounds. In *S. obliquus*, the pyrolysis profile under N + was characterized primarily by esters and reactive intermediates, while under N− it was dominated by hydrocarbons (13.6%) and alcohols. Nitrogen deprivation increased lipid accumulation in all three microalgal species, but the volatile compounds generated during pyrolysis remained species-dependent. Despite the higher lipid content under nitrogen deprivation, the corresponding pyrolysis profiles did not show a consistent relationship with lipid accumulation. These results indicate that lipid-rich biomass does not necessarily exhibit similar thermochemical behavior across microalgal species and suggest that evaluating feedstocks based solely on total lipid content may overlook species-specific differences relevant to bioenergy production.

**Graphical abstract:**

**Figure:**
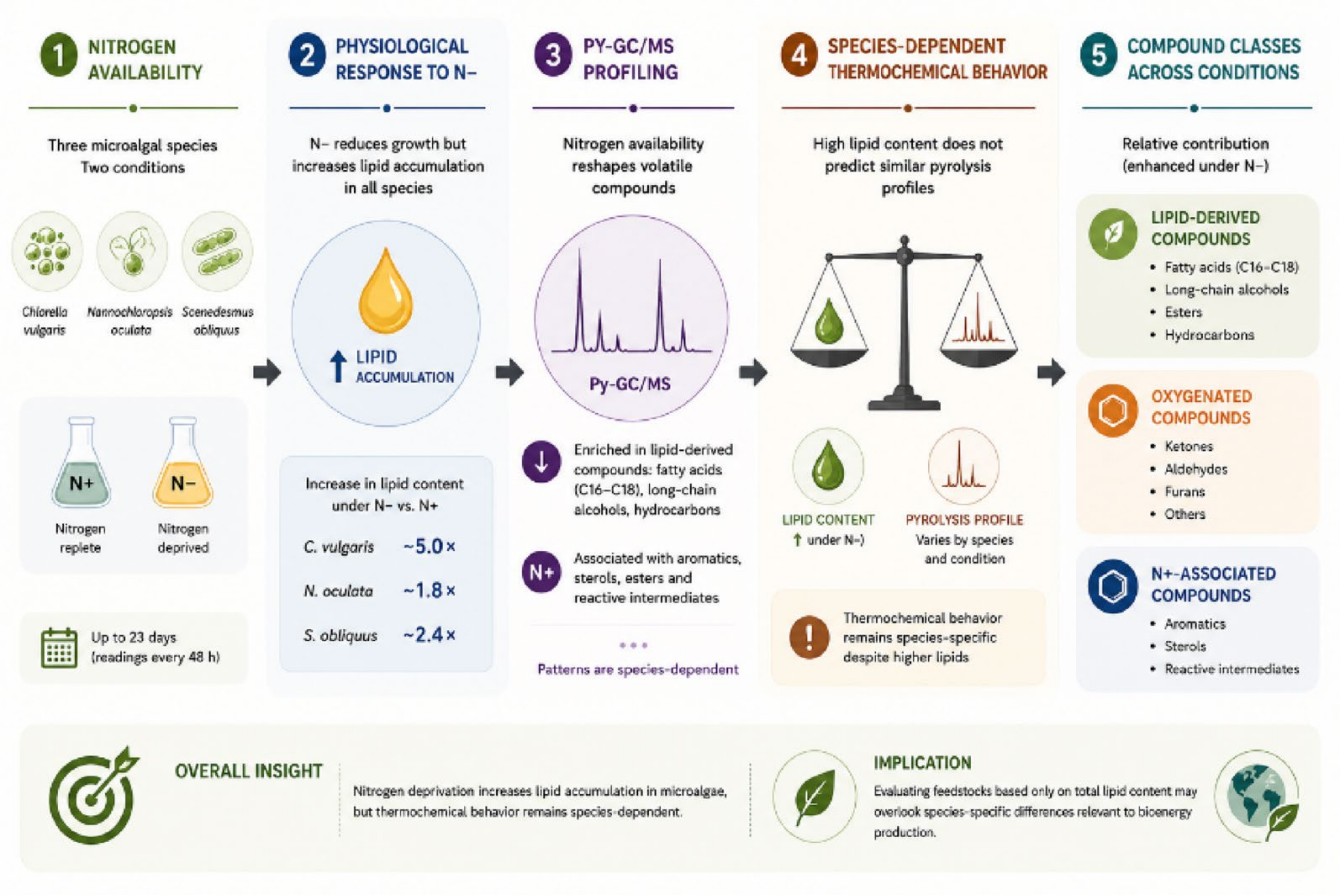

**Supplementary Information:**

The online version contains supplementary material available at 10.1186/s40643-026-01114-4.

## Introduction

Although fossil fuel reserves are gradually declining, they continue to dominate the global energy matrix. Their uneven geographical distribution has heightened concerns about energy security and global stability. Given the negative environmental impacts of fossil fuel use and the high dependence on these resources, they will continue to influence economic volatility and geopolitical tensions in energy markets (Canan et al. 2025). Despite rapid growth in renewable energy capacity, high fossil fuel consumption remains a risk because of its greenhouse gas emissions. Therefore, the development and use of new, cleaner, renewable energy sources are urgently needed (Attanayake et al. 2024).

Biomass, including algae, naturally stores solar energy as chemical energy, making it a sustainable feedstock for energy production with the potential to mitigate environmental impacts and strengthen energy security (Waseem et al. 2026). Currently, various technologies convert the energy stored in biomass into alternative fuels, such as bioethanol, biodiesel, biogas, and biochar. Among thermochemical conversion processes, pyrolysis and gasification have garnered particular interest for their ability to rapidly transform biomass into a range of gaseous, liquid, and solid products (Ullah et al. 2025; Ungureanu et al. 2025). Interest in characterizing the volatile and semi-volatile compounds generated during pyrolysis is growing, as it enables a more detailed understanding of reaction pathways and product distributions that depend on substrate composition (Nair and Verma 2025). Another advantage of the thermochemical conversion of biomass is the ability to use solar concentration systems to supply the thermal energy required for these reactions. This possibility offers two additional benefits: a) efficient storage of solar energy and b) a sustainable pathway for the production of chemical compounds and energy fractions derived from biomass (Kumar et al. 2024).

It has been widely argued that using crops or livestock for energy production can have negative environmental and socioeconomic impacts. Therefore, the most viable biomass sources are those that do not compete with food production, such as agricultural residues, marine biomass (sargassum), and, in particular, microalgae from cultivation systems (Baidoo et al. 2025). Among the various types of biomass, microalgae represent an attractive feedstock for evaluating how nutrient availability (such as nitrogen limitation and/or deprivation) modifies the biochemical composition of the biomass and, consequently, the distribution and energy value of the products obtained in thermochemical conversion (Allen et al. 2018; Hoque et al. 2025; Sadvakasova et al. 2025).

Nitrogen deprivation (N˗) has been the primary strategy for stimulating lipid accumulation in microalgae (Shi et al. 2026; Yang et al. 2024). However, most of these studies have focused on biomass productivity and lipid production, neglecting the analysis of biochemical responses that influence thermochemical conversion processes (Abdelkarim et al. 2025; Narayanan et al. 2025). Therefore, it is necessary to determine whether nitrogen−induced lipid accumulation is accompanied by changes in the composition and distribution of volatile compounds generated during microalgal biomass pyrolysis.

The thermochemical conversion of microalgae via pyrolysis has emerged as a significant route for converting their biochemical components into fuels and chemicals. Due to their high lipid and protein content, as well as their high hydrogen−to-carbon (H/C) ratios, microalgae offer advantages in terms of pyrolysis product yield and quality compared to other lignocellulosic substrates (Alizad Oghyanous and Eskicioglu 2025; de Deus Junior et al. 2024). Recent Py-GC/MS studies have shown that microalgal pyrolysis produces a wide range of volatile compounds, such as fatty acids, long-chain alcohols, hydrocarbons, and aromatic compounds, whose distribution depends on the proportion of lipids, proteins, and carbohydrates in the biomass (Aliyu et al. 2025; Lezcano et al. 2025). Consequently, this composition directly influences the quality and energy potential of the products obtained.

Enzymes involved in fatty-acid and triacylglycerol biosynthesis, including acetyl-CoA carboxylase, fatty-acid synthase components, and different acyltransferases, participate in carbon allocation and lipid formation in microalgae (Liufu et al. 2025). Protein˗protein association networks can provide complementary information about the predicted functional organization of these enzymes when adequate genome annotation is available for the organism of interest in the reference database (Szklarczyk et al. 2019). However, such networks describe predicted functional associations and do not provide direct evidence of enzyme activity or conditioN−dependent regulation. Accordingly, network analysis may be used as a complementary tool to contextualize lipid-biosynthesis pathways, but it must be interpreted separately from the experimentally measured responses to nitrogen deprivation (Panahi and Khalilpour Shadbad 2024). Despite advances in understanding microalgal lipid metabolism and thermochemical conversion, lipid accumulation and pyrolysis-derived products have frequently been examined independently or in individual species (Mathimani et al. 2019). Consequently, it remains unclear whether the increase in lipid content induced by nitrogen deprivation is consistently accompanied by comparable changes in pyrolysis-derived volatile composition across microalgae with different biochemical characteristics (Mustapha et al. 2023; Silva et al. 2016). A comparative evaluation of these responses is therefore needed to better understand the species-specific thermochemical behavior of microalgal biomass (Ağbulut et al. 2023).

In this work, we evaluated biomass growth, lipid accumulation, and pyrolysis-derived volatile composition in three microalgal species of bioenergy interest, *Scenedesmus obliquus*, *Chlorella vulgaris*, and *Nannochloropsis oculata*, cultivated under nitrogen−replete (N +) and nitrogen−deprived (N−) conditions. These responses were compared across species to determine whether nitrogen−induced lipid accumulation was consistently accompanied by changes in pyrolysis-derived volatile composition. In addition, a predicted protein−association network was examined in *S. obliquus*, for which sufficient annotation information was available in STRING, to provide complementary functional context for lipid biosynthesis pathways without inferring condition−dependent regulation.

This study improves the understanding of how nitrogen availability influences lipid accumulation and the pyrolysis-derived volatile composition of microalgal biomass, providing additional insight into its thermochemical behavior. The generated knowledge supports the United Nations Sustainable Development Goals (SDGs) 7 (Affordable and Clean Energy) and 9 (Industry, Innovation and Infrastructure) by contributing to the sustainable development of microalgal bioenergy through improved characterization of biomass with biofuel potential (Fegade 2024a, b).

## Methods

### Culture of *Scenedesmus obliquus*, *Chlorella vulgaris* and *Nannochloropsis oculata*.

The three microalgal species were obtained from the University of Texas Culture Collection of Algae (UTEX) and cultured in sterile Bold’s Basal Medium (BBM) at pH 8. The inoculum was adjusted to an optical density of 0.10 ± 0.01 (measured at 750 nm). Cultures were incubated at 22 ± 2 °C under a 16:8 h light/dark photoperiod with cool-white fluorescent lamps (OSRAM L 36W/850) providing an irradiance of 76 µmol photons m^−2^ s^−1^. Continuous aeration was supplied at 1500 cc min^−1^. Growth was monitored every 48 h by measuring optical density at 750 nm (OD_750_), and total lipid content was determined every 72 h until the early stationary phase was detected. Nitrogen−deprived (N−) cultures were prepared using BBM without NaNO_3_ (0% nitrogen source). Prior to inoculation, cells from the standard BBM pre-culture were harvested by centrifugation, washed with distilled water, and resuspended in the nitrogen−deprived medium. The nitrogen−replete (N +) control cultures were grown in standard BBM supplemented with 100% NaNO_3_. Culture conditions and growth measurements were performed as previously described by Correa-Aguado et al. (2021).

### Total lipid quantification by the sulfo-phospho-vanillin (SPV) method

For lipid quantification, the sulfo-phospho-vanillin (SPV) method described by Mishra et al. (2014) was followed. A lipid standard solution was prepared by dissolving canola oil in chloroform (ACS grade, J.T. Baker) at a concentration of 2 mg mL^−1^ and stored at -20 °C until use. A calibration curve was generated by dispensing known amounts of the standard solution into glass tubes. The chloroform was completely evaporated at 60 °C, after which 100 µL of deionized water was added and the samples were processed according to the SPV assay.

For preparation of the SPV reagent, 0.6 g of vanillin (Sigma-Aldrich) was dissolved in 10 mL of absolute ethanol (ACS grade, J.T. Baker), and then 90 mL of deionized water was added while stirring continuously. Subsequently, 400 mL of concentrated phosphoric acid (H_3_PO_4_) (ACS grade, J.T. Baker) was added. The reagent was stored in the dark at 4 °C until use. For lipid determination, a known amount of microalgal biomass was suspended in 100 µL of deionized water. Concentrated sulfuric acid (H_2_SO_4_) (ACS grade, J.T. Baker) was then added, and the samples were heated at 100 °C for 10 min, followed by cooling in an ice bath for 5 min. Later, 5 mL of freshly prepared phospho-vanillin reagent was added, and the samples were incubated at 37 °C for 15 min with occasional shaking. Finally, absorbance was measured at 530 nm, and lipid content was quantified using the calibration curve.

### Gravimetric validation of the SPV method

To validate the lipid content determined by the sulfo-phospho-vanillin (SPV) assay, total lipids were independently quantified using a gravimetric extraction method. Briefly, freeze-dried biomass (10 mg) from each of the three microalgal species was mixed with 600 μL of chloroform:methanol (2:1, v/v) (ACS grade, J.T. Baker), vortexed for 2 min, and centrifuged at 13,000 rpm for 15 min. The extraction was repeated three times for each microalgal species. The collected supernatants were pooled and dried at 50 °C. Total lipid content was determined gravimetrically, and all experiments were performed in triplicate (Byreddy et al. 2016).

### Preparation of biomass for analytical pyrolysis

The microalgal biomass was separated by centrifugation at 6500 rpm for 15 min, washed three times with deionized water, and dried at 60 °C for 24 h until a constant weight was reached. The dried biomass was stored at -20 °C until analytical pyrolysis (Mohammed et al. 2018).

### Analytical pyrolysis coupled to gas chromatography–mass spectrometry (Py-GC/MS)

Analytical pyrolysis was performed using a simultaneous thermal analyzer (STA 449 F5 Jupiter, Netzsch, Germany) coupled to a gas chromatography-mass spectrometry (GC–MS) system. Dried biomass (5 mg; particle size approximately 0.2 mm, *n* = 3) was placed in Al_2_O_3_ crucibles and heated from room temperature to 800 °C at a constant heating rate of 20 °C min^−1^ under a helium atmosphere (20 mL min^−1^). The volatile products released during pyrolysis were transferred through a heated transfer line (TRG 004, Netzsch) maintained at 300 °C into an Agilent 7820A gas chromatograph coupled to a 5977B Mass Selective Detector (Agilent Technologies, USA). Compound separation was performed using an HP-5MS capillary column (30 m × 0.25 mm i.d. × 0.25 μm film thickness; 5% phenyl-methylpolysiloxane).

The GC oven was maintained at 250 °C throughout the analysis, with a 5 min solvent delay. The MS ion source temperature was maintained at 150 °C. Compounds were identified by comparison with the National Institute of Standards and Technology (NIST) mass spectral library (Gaithersburg, MD, USA). Only matches with spectral similarity values greater than 80% were accepted.

### Protein−association network construction and analysis

A predicted functional protein−association network was generated for *Scenedesmus obliquus* using STRING (Search Tool for the Retrieval of Interacting Genes/Proteins; version 12.0) (Szklarczyk et al. 2025).

The input set comprised selected proteins involved in fatty acid biosynthesis and glycerolipid assembly, including acetyl-CoA carboxylase, acyl carrier protein, ketoacyl-ACP synthase, and various acyltransferases (Table 1). The proteins were submitted to STRING as query proteins, excluding first- and second-shell interactors. A high-confidence interaction threshold (STRING score ≥ 0.700) was applied. The active evidence channels were text mining, experiments, curated databases, and co-expression. No additional first- or second-shell interactors were incorporated; therefore, the analysis was restricted to the submitted proteins that could be mapped in STRING. The resulting network was examined descriptively to identify predicted functional associations among the selected lipid-related proteins. STRING scores were interpreted as confidence estimates for functional protein associations rather than as measures of interaction strength, protein abundance, enzymatic activity, or nitrogen−dependent regulation.

**Table 1:** Lipid-biosynthesis proteins selected as input for the predicted protein−association network of *S. obliquus*

Protein/function	Abbreviation	Relevance to lipid metabolism	References
Biotin carboxylase	BC	Catalytic component of acetyl-CoA carboxylase, which participates in malonyl-CoA formation during de novo fatty-acid biosynthesis	Kruse et al. (2025); Mavila et al. (2025)
Biotin carboxyl carrier protein	BCCP	Carrier component of the acetyl-CoA carboxylase complex involved in malonyl-CoA formation	
Acyl carrier protein	ACP	Carries acyl intermediates during de novo fatty-acid biosynthesis	Gwak et al. (2014); Song et al. (2024)
β-Ketoacyl-ACP synthase	KAS	Catalyzes successive condensation reactions involved in fatty-acid-chain elongation	
Glycerol-3-phosphate acyltransferase	GPAT	Catalyzes the first acylation step of glycerolipid biosynthesis, producing lysophosphatidic acid (LPA)	Zou et al. (2023)
1-Acyl-sn−glycerol-3-phosphate acyltransferase	LPAAT	Catalyzes the second acylation step of glycerolipid biosynthesis, producing phosphatidic acid (PA), a common precursor of membrane and storage lipids	Yamaoka et al. (2016)

## Results

### Growth dynamics of microalgae under nitrogen−replete and nitrogen−deprived conditions

**Fig. 1:**
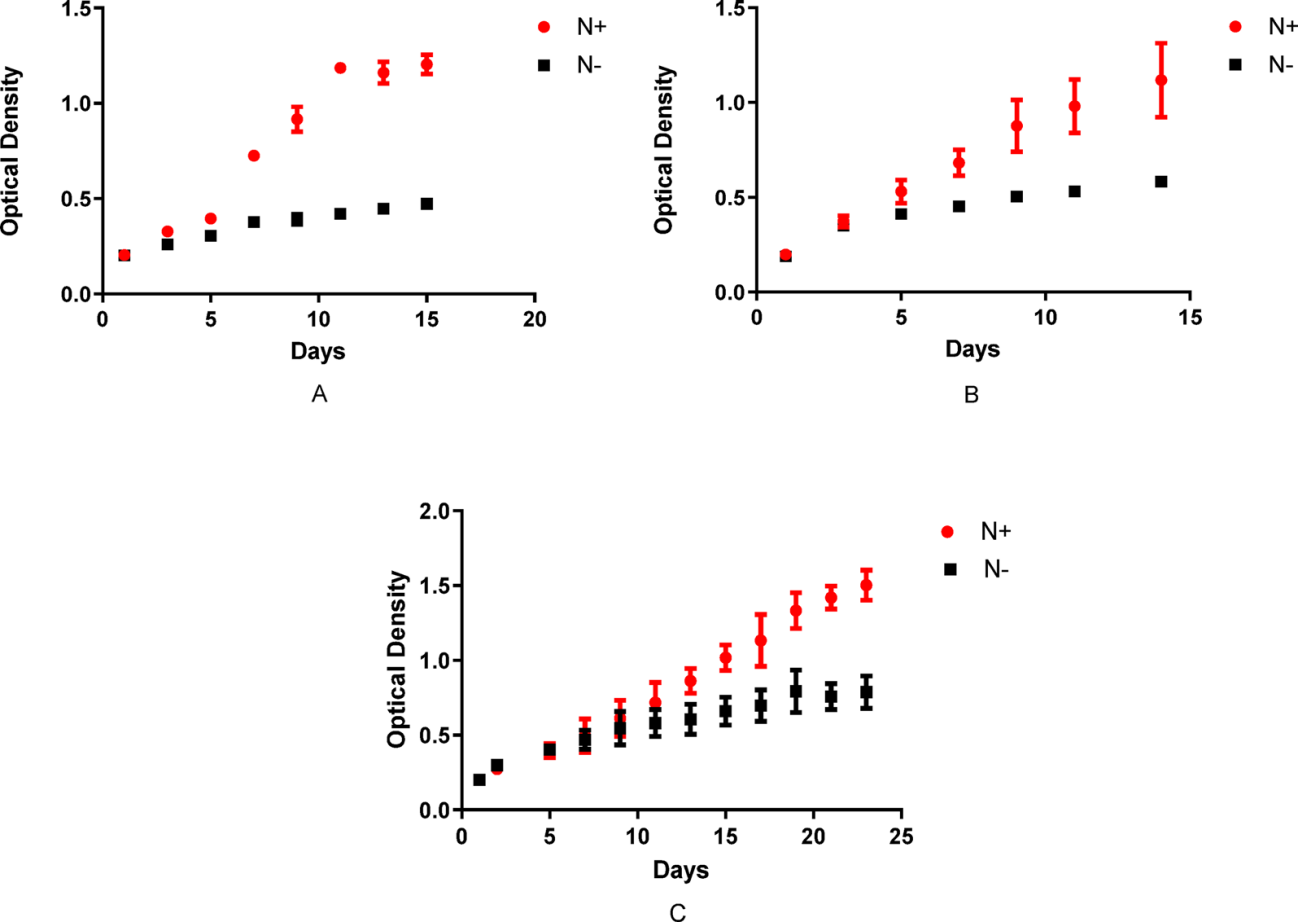
Growth kinetics of **A** *S. obliquus*, **B** *C. vulgaris*, and **C** *N. oculata* under nitrogen−replete (N +) and nitrogen−deprived (N−) conditions. Optical density (OD_750_) was measured every 48 h. Data represent mean ± SD (*n* = 3). The duration of cultivation was determined by detecting the onset of the early stationary phase under N˗ conditions for each species

*C. vulgaris*, *N. oculata*, *and S. obliquus* grew under both N + and N − conditions. However, nitrogen deprivation reduced growth in all three species, resulting in consistently lower optical density values under N− throughout cultivation (Fig. 1). Despite the lower biomass accumulation, sufficient biomass was obtained for subsequent biochemical and thermochemical analyses.

### Lipid accumulation in microalgae under N + and N− conditions

**Fig. 2:**
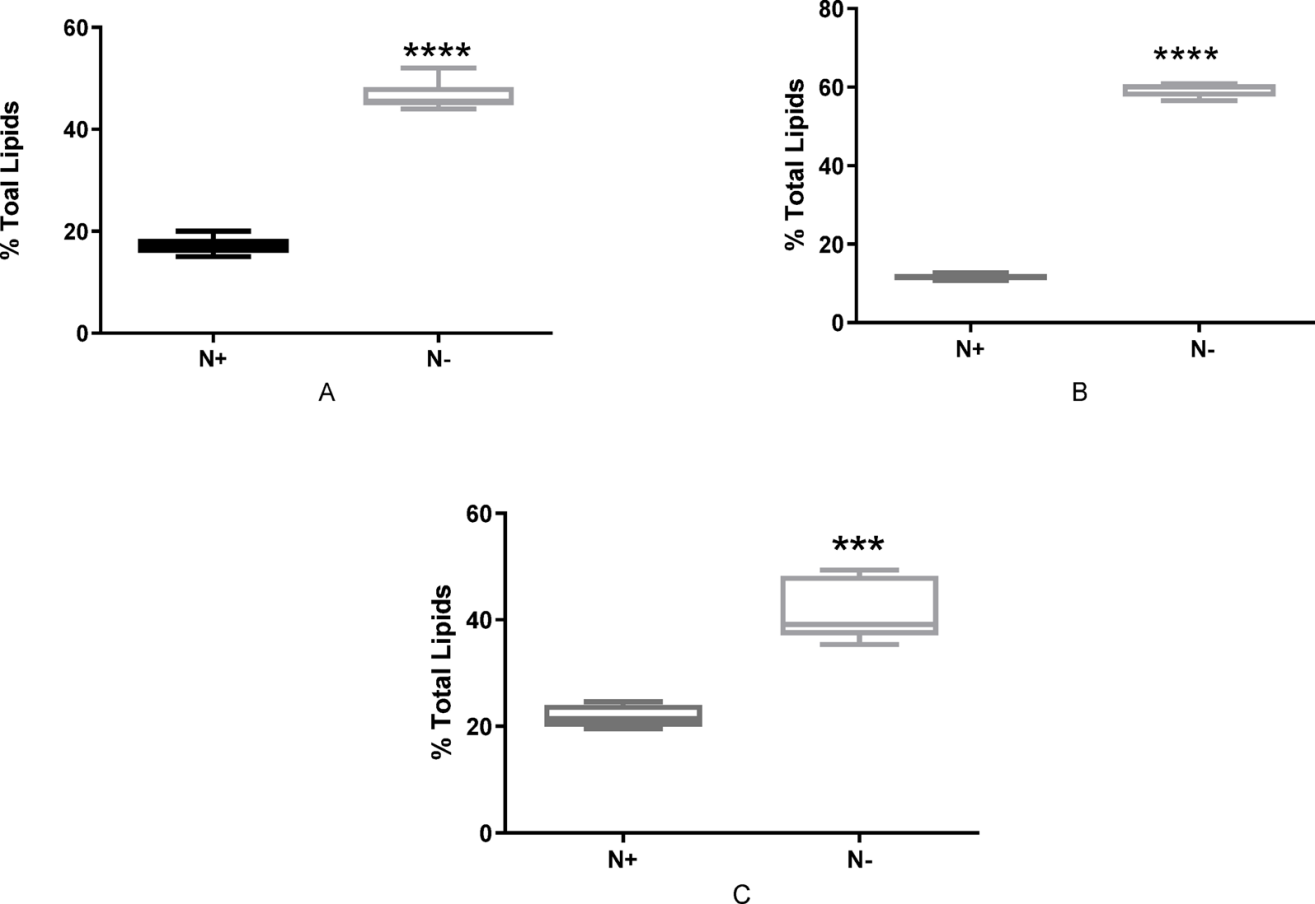
Total lipid content (% dry weight) of **A** *S. obliquus*, **B** *C. vulgaris*, and **C** *N. oculata* under N−replete (N +) and N−deprived (N˗) conditions. Data are presented as mean ± SD (*n* = *3*). Differences between N + and N− conditions were evaluated using Student’s t-test. Statistical significance is indicated by asterisks (***p < 0.001; **p < 0.0001)

Nitrogen deprivation enhanced lipid accumulation in *S. obliquus*, *C. vulgaris*, and* N. oculata* species (*p* < 0.05). Figure 2 shows that the total lipid content of *C. vulgaris* increased markedly from 12 to 60% under N−conditions, representing a 5.0-fold increase and the highest relative lipid enrichment among the evaluated species. Similar increases in total lipids were observed in *S. obliquus* (2.4-fold) and *N. oculata* (1.8-fold). Also, it was observed that proliferation of N + cells was faster than that of N− cells (Fig. 1).

### Pyrolysis-derived volatile compounds of microalgae under N + and N− conditions

Py-GC/MS revealed differences in the composition and relative distribution of volatile compounds among the three microalgal species and between N + and N− conditions. The major compounds identified are summarized in Table 2.

**Table 2:** Major volatile compounds (≥ 7.5% GC–MS relative area) from microalgae pyrolysis under nitrogen−replete (N +) and nitrogen−deprived (N−) conditions

Microalgae species	Nitrogen condition	Major compound	Relative area (%)	Chemical class
*Chlorella vulgaris*	N−	Oleic acid	22.3	Fatty acid
		2-methyl-1-hexadecanol	7.9	Alcohol
		3,7,11-trimethyl-1-dodecanol	7.5	Alcohol
	N +	17-Octadecynoic acid	12.6	Fatty acid
		10-HeptadeceN−8-ynoic acid, methyl ester	11.1	Ester
		(3-octylundecyl)-benzene	8.8	Aromatic
*Nannochloropsis oculata*	N +	17-Octadecynoic acid	26.3	Fatty acid
		Neophytadiene	22.2	Hydrocarbon
		9,12-Octadecadienoyl chloride	14.1	Acyl chloride
	N−	Oleic acid	16.9	Fatty acid
		17-Octadecynoic acid	13.1	Fatty acid
		2-Furanmethanol	8.8	Oxygenated
*Scenedesmus obliquus*	N +	10-HeptadeceN−8-ynoic acid, methyl ester	25.3	Ester
		9,12-Octadecadienoyl chloride	20.5	Acyl chloride
	N−	1,4,6,9-Nonadecatetraene	13.6	Hydrocarbon
		Oleic acid	11.6	Fatty acid
		1-Dodecanol, 3,7,11-trimethyl-	9.6	Alcohol

See Supplementary Data S1 for the complete list of detected compounds

The resulting volatile fraction of *C. vulgaris* (N−) biomass consisted largely of C16-C18 lipid-derived species.

On the other hand, the thermochemical treatment of *C. vulgaris* N + biomass exhibited a more heterogeneous composition. The main compounds were derived from C18 fatty acids (11˗12%), aromatic compounds (5–8%), and sterol-type compounds (5%). No evidence was observed that a specific type of free fatty acid dominated the profile of volatile compounds under these conditions (N +).

Similarly, the pyrolytic derivatives of *N. oculata* biomass (N + and N−) showed a volatile profile dominated by lipid-derived C16-C18 compounds. Under N + conditions, the dominant compounds were 17-octadecenoic acid (26.31%) and neophytadiene (22.21%). Under N− conditions, various volatile compounds were obtained. For example, compounds derived from long-chain alcohols and oxygenated compounds were detected, and, in general, derivatives of modified C18 fatty acids were observed. The most abundant compound was oleic acid (16.9%), followed by 17-octadecenoic acid and the methyl ester of 10-heptadecen−8-enoic acid. In addition, oxygenated compounds, such as 2-furanomethanol, were detected. Unlike *C. vulgaris*, nitrogen deprivation in *N. oculata* was associated with a redistribution of volatile compounds, although a predominantly oily and aliphatic character was maintained (Table 2).

Regarding *S. obliquus,* the decomposition profile under N + treatment was more complex than those of *C. vulgaris* and *N. oculata*. For example, derivatives of unsaturated fatty acids, such as 10-heptadecene-8-ynoic acid methyl ester and 17-octadecenoic acid, were detected. In addition, reactive intermediate compounds such as 12-octadecadienoyl chloride and 10-undecenoyl chloride were obtained. In contrast, the N− treatment in *S. obliquus* showed a profile of non−oxygenated aliphatic lipid-derived volatile products. The most abundant compounds were 1,4,6,9-nonadecatetraene, oleic acid, 3,7,11-trimethyl-1-dodecanol, and 2-methyl-1-hexadecanol. Other hydrocarbon species, such as 4,6,9-nonadecatriene, also contributed to the volatile profile (Table 2).

Overall, the volatile profiles under N− showed a greater contribution of lipid-derived compounds, primarily C16-C18 fatty acids, long-chain alcohols, and aliphatic hydrocarbons.

**Fig. 3:**
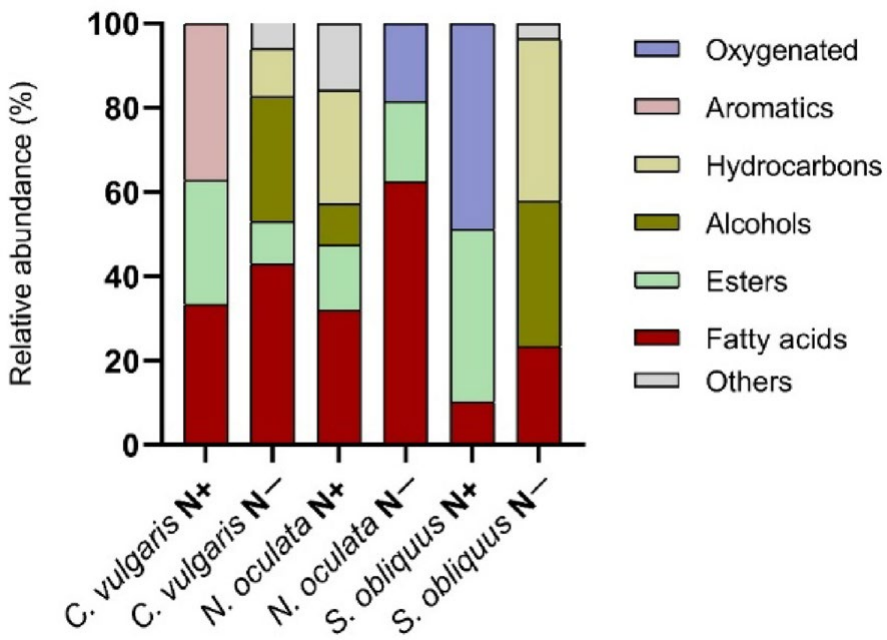
Distribution of volatile compound classes during microalgal pyrolysis under nitrogen−replete (N +) and nitrogen−starved (N−) conditions. Values represent the relative abundance of major compound classes identified by GC–MS. Minor (< 5%) were grouped as “Others”

Figure 3 shows the distribution of volatiles obtained through the pyrolysis of microalgae. The volatile fractions were classified into the following compound types: fatty acids, esters, alcohols, hydrocarbons (saturated and unsaturated), aromatics, and oxygenated compounds, including ketones, aldehydes, carboxylic acids, and related derivatives. Under N− conditions, the volatile profile of *C. vulgaris* contained higher proportions of fatty acids and alcohols than under N + conditions. Regarding *N. oculata*, fatty acids were the predominant fraction, especially under N˗, whereas under N + , a more balanced distribution of compounds was observed. Finally, the *S. obliquus* volatile profile contained higher proportions of esters and oxygenated compounds in N + , whereas fatty acids, alcohols, and hydrocarbons contributed more strongly under N− conditions.

### Predicted lipid-related protein−association network in *Scenedesmus obliquus*

**Fig. 4:**
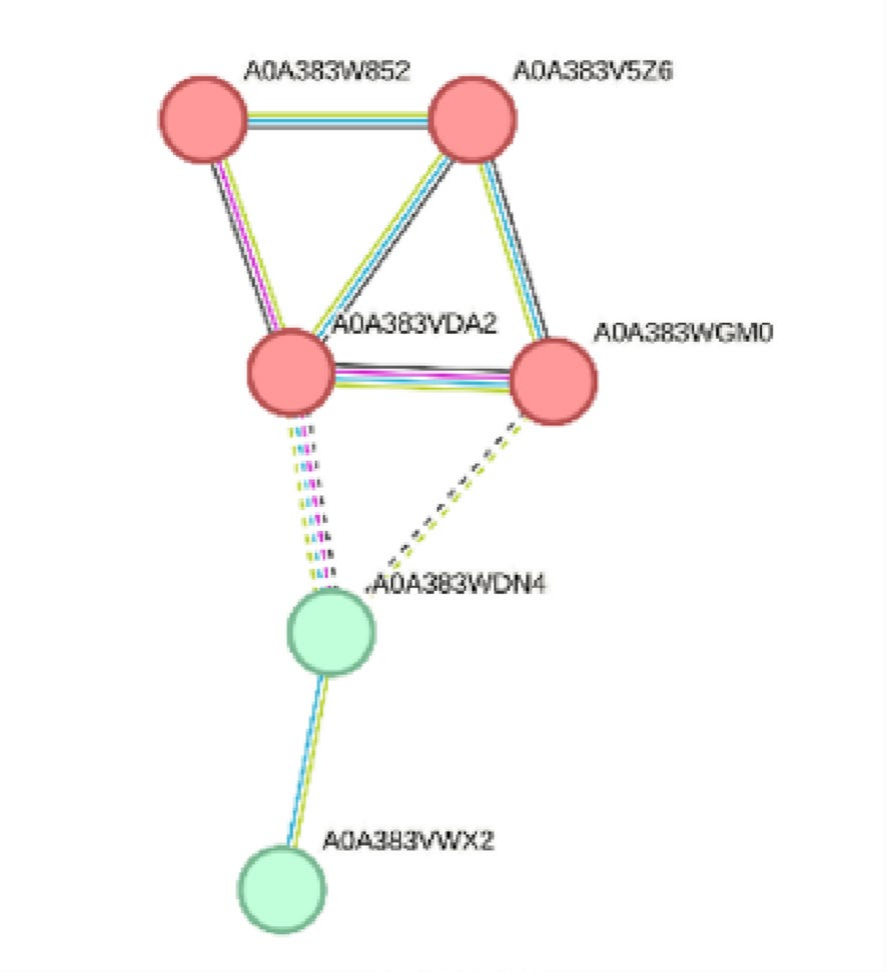
Predicted functional protein−association network of selected lipid-related proteins in *Scenedesmus obliquus*. The network was generated in STRING using six predefined proteins involved in fatty acid biosynthesis and glycerolipid assembly, without adding first- or second-shell interactors. Nodes represent the submitted proteins, and edges represent predicted functional associations supported by the selected STRING evidence channels at a minimum confidence score of 0.700. Edge colors indicate the different sources of evidence contributing to each association. The network provides a reference representation of predicted functional relationships and does not represent condition−dependent protein activity or regulation

STRING analysis of the predefined lipid-related proteins produced a predicted functional protein−association network for *S. obliquus* (Fig. 4). At the selected confidence threshold (score ≥ 0.700), BC, BCCP, ACP, and KAS formed a connected group comprising proteins associated with de novo fatty-acid biosynthesis. GPAT and LPAAT showed fewer associations with this group and occupied more peripheral positions in the network. No additional first- or second-shell interactors were included. The resulting network describes predicted functional associations among the selected proteins and does not represent experimentally measured protein activity or nitrogen−dependent regulation.

**Fig. 5:**
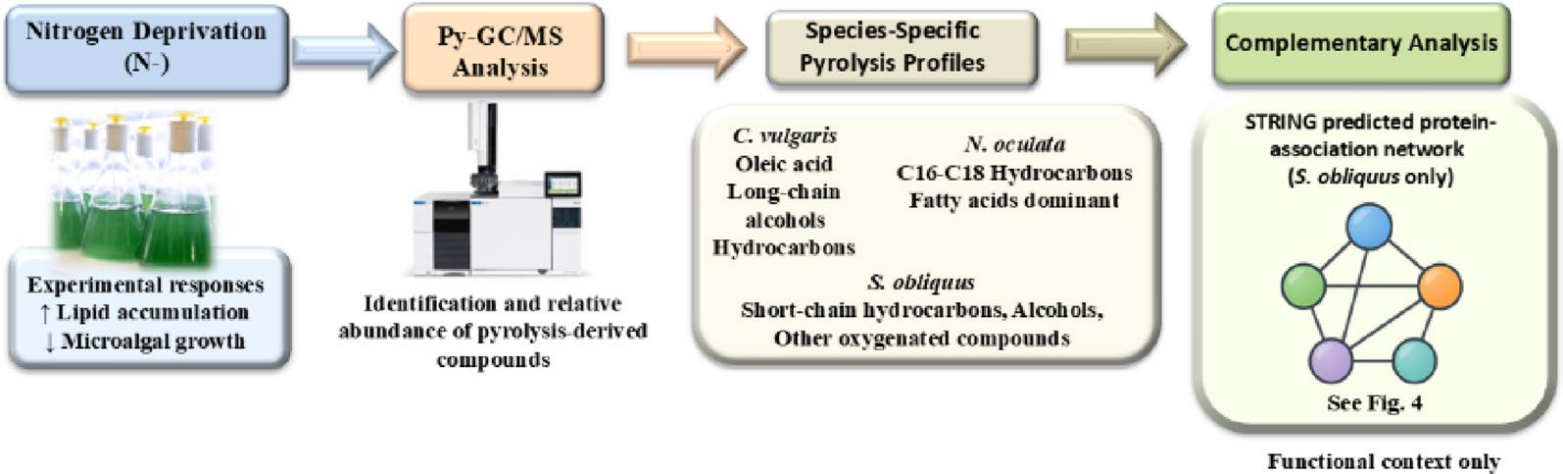
Overview of the experimental workflow. Three microalgal species were cultivated under nitrogen deprivation (N−) to evaluate lipid accumulation, microalgal growth, and the relative abundance of pyrolysis-derived compounds by Py-GC/MS. Representative compounds identified under N− are shown. A complementary predicted protein−association network analysis was performed for *S. obliquus*

Finally, Fig. 5 summarizes the main experimental findings under nitrogen−deprived conditions, including lipid accumulation and the species-specific distribution of pyrolysis-derived volatile compounds, together with the complementary predicted protein−association network for *S. obliquus*. Under N−, lipid accumulation increased in all three species, and differences in pyrolysis-derived volatile compounds were observed among species.

## Discussion

Nitrogen deprivation reduced the growth of all three microalgal species (Fig. 1A–C), although the degree of inhibition differed among species. In contrast, lipid accumulation increased under N−, reaching 60% in *C. vulgaris*, 45% in *S. obliquus*, and 39% in *N. oculata* (Fig. 2A–C). These results agree with previous reports demonstrating that N deprivation reduces cell division and proliferation by reducing protein and nucleic acid synthesis (Maltsev et al. 2023; Şirin and Serdar 2024). Likewise, Barrera‐Duarte et al. (2026) observed growth inhibition accompanied by increased TAG accumulation, and Shi et al. (2026) reported transcriptional changes associated with carbon redistribution during nitrogen stress.

The lipid accumulation varied among *C. vulgaris*, *N. oculata,* and *S. obliquus*, indicating that the response to nitrogen deprivation was species dependent (Fig. 2A–C). This behavior is consistent with that reported for strains of *Chlorella sorokiniana* (CsDOE1412 and Cs1228), which exhibited varying levels of TAG accumulation, even under identical N−limited conditions. The CsDOE1412 strain accumulated 40%, while Cs1228 accumulated 24%.

Despite the increase in lipid content under N−, the three microalgae exhibited distinct pyrolysis-derived volatile profiles. Oleic acid predominated in *C. vulgaris*. In *N. oculata*, lipid-derived compounds remained predominant, with oleic acid and 17-octadecynoic acid as the major products, along with a greater contribution from oxygenated compounds. By contrast, the volatile profile of *S. obliquus* was dominated by hydrocarbons, fatty acids, and long-chain alcohols. These observations suggest that increased lipid accumulation did not translate into equivalent thermochemical behavior across species.

Previous studies have shown that nitrogen deprivation promotes the accumulation of neutral lipids, primarily triacylglycerides (TAG), as an adaptive response to nutrient limitation (Cui et al. 2024). Similar increases have been reported for *Scenedesmus* sp. CABeR52, which exhibited a twofold increase in lipid content under nitrogen deprivation (Abrha et al. 2025). *N. oculata* is a species recognized for maintaining relatively stable lipid production through carbon allocation toward storage compounds under nutrient stress (Navalho et al. 2025). Consistent with these mechanisms, Py-GC/MS analysis in the present study revealed higher relative abundances of lipid-derived compounds, particularly oleic acid and long-chain esters, in *C. vulgaris* and *N. oculata* (Table 2), consistent with the contribution of TAG-rich biomass to the generation of fatty acid-derived pyrolysis products (Chu et al. 2019; Suparmaniam et al. 2023).

Py-GC/MS revealed distinct species-specific volatile profiles under nitrogen deprivation (Table 2). Oleic acid predominated in *C. vulgaris* biomass (22.3%), *N. oculata* was characterized by higher relative abundances of 17-octadecynoic acid and neophytadiene. In contrast, *S. obliquus* showed a greater proportion of oxygenated compounds, consistent with differences in lipid decomposition and secondary pyrolytic reactions (López-Aguilar et al. 2022). These observations are consistent with previous reports showing that biomass biochemical composition, rather than lipid content alone, influences the distribution of pyrolysis-derived products (Kebelmann et al. 2013; Lezcano et al. 2025).

Fatty acids such as oleic acid, along with alcohols and long-chain hydrocarbons, were predominantly detected under N−, particularly in *C. vulgaris* and *S. obliquus*. This observation is consistent with a greater contribution of lipid-derived precursors to the volatile profile identified after pyrolysis. In contrast, fatty acid esters were more frequently detected under nitrogen−replete (N +) conditions, suggesting that distinct pyrolysis pathways may predominate depending on biomass composition and cultivation conditions.

**Fig. 6:**
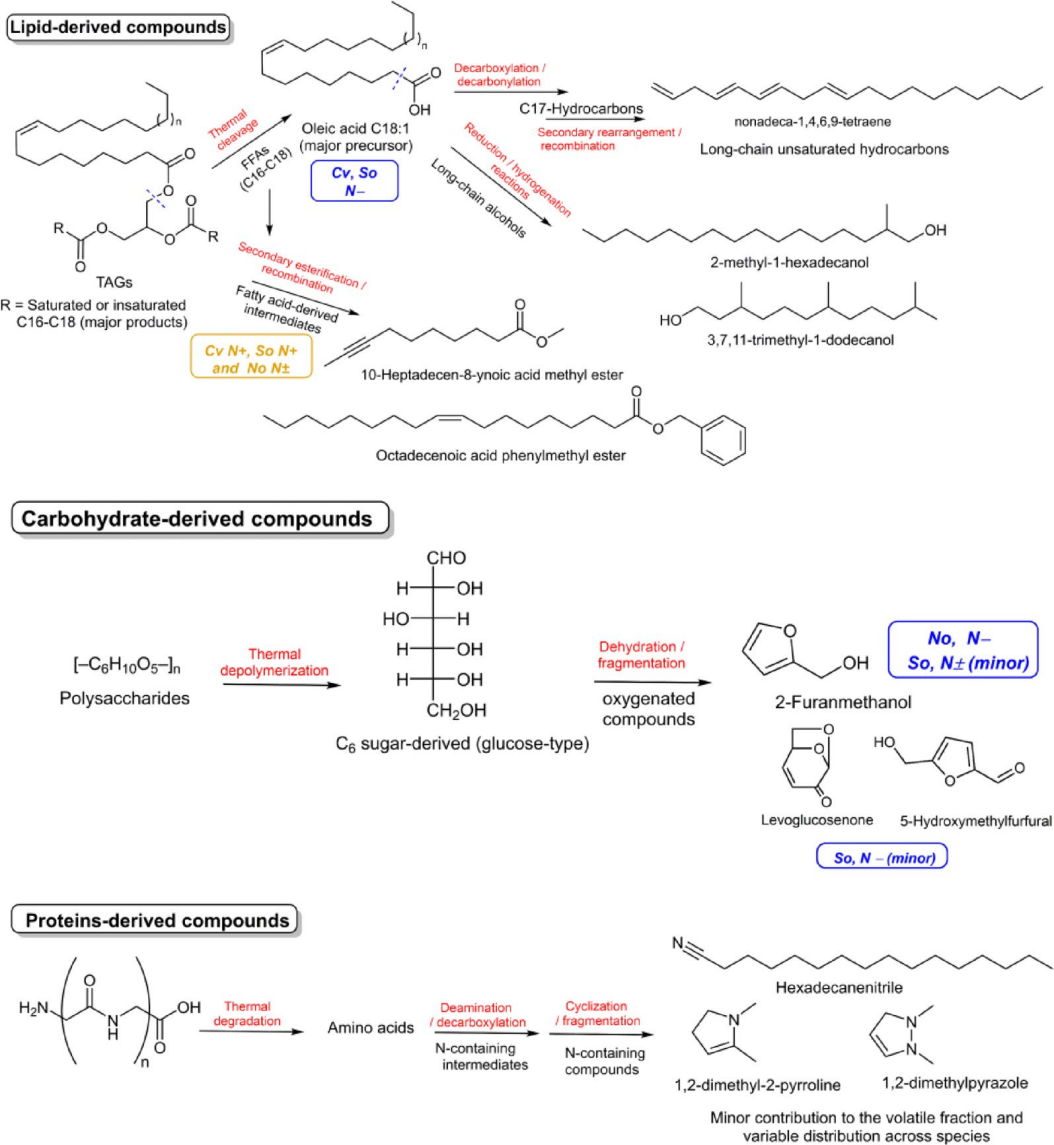
Proposed pyrolytic pathways of lipid-, carbohydrate-, and protein−derived compounds in microalgae (*Cv: Chlorella vulgaris*, *No: Nannochloropsis oculata* and *So: Scenedesmus obliquus*) under different nitrogen conditions (N−, nitrogen−deprived; N + , nitrogen−replete). The scheme highlights the dominant role of lipid-derived pathways and the secondary contributions of carbohydrate and protein fractions in shaping the distribution of pyrolysis products

Figure 6 illustrates the proposed fragmentation pathways of the main compounds identified by Py-GC/MS. The predominance of C16-C18 fatty acids, particularly oleic acid, validates their role as key precursors in the distribution of pyrolysis-derived products. These fatty acids are considered to undergo thermal degradation primarily via decarboxylation and decarbonylation, yielding hydrocarbons and alcohols (Wang et al. 2025). The formation of long-chain alcohols may also reflect secondary pyrolytic transformations involving oxygenated lipid intermediates. The absence of certain compounds in specific species, such as long-chain hydrocarbons and alcohols in *N. oculata*, is consistent with the species-dependent nature of pyrolytic pathways and highlights the role of biochemical composition in determining product distribution.

Although lipid-derived compounds dominated the volatile fraction, minor contributions from carbohydrate- and protein−derived pathways were also identified. Carbohydrate-derived products were detected primarily in *N. oculata* under n− conditions, where 2-furanmethanol was the most abundant oxygenated compound. Its formation is consistent with the thermal degradation of polysaccharides via depolymerization and dehydration of sugar-derived intermediates, yielding furan−type oxygenated compounds (Carrasco Díaz et al. 2024; Osatiashtiani et al. 2022).

The proposed formation of additional compounds, including levoglucosenone and 5-hydroxymethylfurfural (Fig. 6), further illustrates the potential contribution of secondary carbohydrate-derived pathways during pyrolysis, consistent with previous reports showing that carbohydrate-rich microalgal biomass generates higher proportions of oxygenated volatile compounds during thermochemical conversion (Bach and Chen 2017; Patwardhan et al. 2009; Widawati et al. 2025).

In line with our observations, other studies have shown that under nitrogen stress, microalgae reduce protein and chlorophyll synthesis and favor the accumulation of reserve compounds that serve as precursors for various volatile products in thermochemical processes such as pyrolysis (Khoo et al. 2023; Narayanan et al. 2025). The protein−derived compounds exhibited low and heterogeneous distribution across species, precluding the identification of a clear species-specific pattern. Nitrogen−containing compounds such as nitriles and heterocyclic structures, including pyrroline and pyrazole derivatives, were identified, consistent with protein degradation pathways involving deamination, decarboxylation, and cyclization reactions (Hu et al. 2025; Huang et al. 2017). The lipid-, carbohydrate-, and protein−derived volatile profiles indicate that N availability modifies not only the relative abundance of biomass constituents but also the diversity of thermochemical reaction products generated during pyrolysis. The species-dependent distribution of these compounds suggests that the biochemical composition of microalgae influences thermochemical conversion through multiple interconnected degradation pathways rather than through a single dominant precursor pool.

A predicted functional protein association network for *S. obliquus* was examined in silico. The network showed associations among BC, BCCP, ACP, and KAS, which participate in de novo fatty acid biosynthesis, whereas GPAT and LPAAT showed fewer associations with this group. This organization is consistent with the established biochemical roles of these enzymes in fatty-acid and glycerolipid biosynthesis (Gwak et al. 2014; Yamaoka et al. 2016; Zou et al. 2023). However, the network was generated from a predefined set of proteins and did not incorporate gene expression, protein abundance, or enzyme activity measurements from the N + and N− treatments. Consequently, it provides only a reference description of predicted functional associations in *S. obliquus* and cannot be used to infer nitrogen−dependent regulation or explain the differences in pyrolysis-derived compounds among the three species.

Nitrogen deprivation reduced microalgal growth while promoting species-dependent lipid accumulation and changes in pyrolysis-derived volatile composition. Although lipid-derived compounds accounted for a significant fraction of the volatile products, the three species did not exhibit identical thermochemical profiles. This finding indicates that total lipid content alone is insufficient to predict the distribution of pyrolysis products and that differences in fatty acid composition and in the relative contributions of lipid-, carbohydrate-, and protein−derived precursors must also be considered.

Unlike recent studies, which have focused primarily on the thermochemical characterization of microalgal biomass and the distribution of pyrolysis-derived compounds (da Silveira Rossi et al. 2023; Yang et al. 2025), the present study combines physiological measurements, lipid accumulation, and Py-GC/MS analysis to compare species-specific responses to nitrogen deprivation. A complementary predicted protein−association network was included only to provide functional context for lipid biosynthesis in *S. obliquus* and was not used to infer nitrogen−dependent regulation. The predominance of fatty acid- and hydrocarboN−derived compounds further supports the bioenergy potential of microalgal biomass (Elsafi et al. 2025).

Integrating biochemical characterization with Py-GC/MS analysis provides a more comprehensive basis for evaluating microalgal biomass for thermochemical conversion and biofuel applications. By supporting informed feedstock selection based on biochemical and thermochemical characteristics, this approach aligns with green engineering and green chemistry principles by using renewable feedstocks and integrating complementary methods for sustainable biofuel development (Anastas and Eghbali 2010; Fegade 2024c).

## Conclusions

Nitrogen availability influenced microalgal growth, lipid accumulation, and the composition of pyrolysis-derived volatile compounds, although the magnitude and nature of these responses differed among the three species. Under nitrogen−deprived conditions, lipid content increased from 12 to 60% in *C. vulgaris* (5.0-fold), from 19 to 45% in *S. obliquus* (2.4-fold), and from 22 to 39% in *N. oculata* (1.8-fold).

Pyrolysis-derived volatile profiles also differed between N + and N− conditions. Under N−, *C. vulgaris* showed higher relative contributions of C16-C18 fatty acids and long-chain alcohols, and *N. oculata* revealed increased oleic acid and oxygenated compounds. In contrast, *S. obliquus* exhibited a shift from esters and acyl chlorides under N + to hydrocarbons, fatty acids, and long-chain alcohols under N−. These differences indicate that nitrogen availability is associated with species-specific differences in the distribution of pyrolysis-derived products.

Overall, increased lipid content did not yield equivalent pyrolysis-derived volatile profiles among the three microalgae. Therefore, total lipid content alone is insufficient to characterize the thermochemical behavior of microalgal biomass. Integrating lipid-content measurements with the characterization of lipid-, carbohydrate-, and protein−derived pyrolysis products provides a more comprehensive basis for evaluating microalgal biomass for thermochemical conversion and biorefinery applications.

## Supplementary Information

Below is the link to the electronic supplementary material.

Supplementary Material 1

## Data Availability

All data generated or analyzed during this study are included in this published article and its supplementary information file.
